# Diagnostic accuracy of resistive index of capsular and intratesticular branches of testicular arteries in infertile men with oligoasthenospermia: A case-control study

**DOI:** 10.37796/2211-8039.1036

**Published:** 2020-12-01

**Authors:** Mehdi Zolfaghar-Khani, Hadi Majidi, Behzad Feizzadeh, Mojtaba Sabaghi

**Affiliations:** a Department of Radiology, Faculty of Medicine, Orthopedic Research Center, Mazandaran University of Medical Sciences, Sari, Iran; b Department of Urology, Faculty of Medicine, Mazandaran University of Medical Sciences, Sari, Iran

**Keywords:** Resistive Index, Infertility, Oligoasthenospermia, Color Doppler ultrasound

## Abstract

**Introduction:**

Male infertility accounts for nearly 50% of couples' infertility. Only a few studies evaluate the diagnostic accuracy of resistive index (RI) of testicular arteries using color Doppler imaging to identify male infertility. The aim of this study was to investigate the diagnostic accuracy of RI of the capsular and intratesticular branches of testicular arteries in infertile men with oligoasthenospermia and its comparison with normal men.

**Material and methods:**

In a case-control study, 30 patients with oligoasthenospermia (case group) and 30 healthy controls who meet the inclusion criteria, were selected. For all patients, RI was measured using color Doppler ultrasonography in upper and lower testicular poles. Also, testicular volumes were measured for all participants.

**Results:**

Mean RI of the intratesticular artery (0.624 ± 0.051 versus 0.509 ± 0.054; P < 0.001) and capsular artery (0.663 ± 0.057 versus 0.557 ± 0.055; P < 0.001) were significantly higher in the case group compared to control group. The frequency of abnormal RI of intratesticular artery in the control (13.3%) and case (66.7%) groups were significantly different (OR: 13.0; 95% CI: 3.44-47.59; P < 0.001). The sensitivity, specificity, positive predictive value (PPV), negative predictive value (NPV) and overall accuracy (OA) of intratesticular artery RI were 66.67%, 86.67%, 83.33%, 72.22%, and 76.67%, respectively. The frequency of abnormal RI of capsular artery was 23.3% in the control group and 90.0% in the case group (OR: 29.57; 95% CI: 6.85-127.63; P < 0.001). The sensitivity, specificity, PPV, NPV and OA of capsular artery RI were 90.0%, 76.67%, 79.41%, 88.46% and 83.33%, respectively.

**Conclusion:**

The results of this study indicated that assessing testicular Doppler RI of capsular and intratesticular branches of the testicular arteries, as a bio-imaging biomarker, may be a valuable non-invasive and simple complementary diagnostic modality with a high diagnostic value in identifying infertile men with oligoasthenospermia.

## 1. Introduction

Infertility is defined as couples' inability to conceive following a year of unprotected sexual intercourse [1-2]. Infertility is a prevalent disorder among the reproductive-aged couples that affects approximately 15% of them [3-6]. Male infertility, alone or in combination with female infertility, is attributed to 50% of all causes of infertility [7-10]. There are various diagnostic modalities for male infertility. Due to technological advances in ultrasonography, the role of sonography in evaluating male infertility is expanding. Structural and functional evaluation of testicular tissue, including testicular volume, echogenicity, tissue elasticity as well as testicular macrovascular and microvascular blood flow can be evaluated using ultrasonography. [11-14]. It has been previously confirmed a strong relationship between testicular volume and function [15-17]. Additionally, 70-80% of testicular volume is composed of seminiferous tubules, which are the site of spermatogenesis. Also, it has been shown that in infertile men there is a correlation between their testicular volume and semen profile [11, 18].

Resistive Index (RI) is a recently introduced ultrasonic parameter showing testicular parenchymal perfusion and microcirculation function. This index is measured using S-D/S formula, where S represents the peak systolic velocity (PSV) and D indicate the end diastolic velocity (EDV). The increase in this index in the testicles indicates disruption in micro-circulation and thus a significant reduction in testicular blood flow [19-22]. As spermatogenesis is a highly sensitive and precise process where blood demand and supply are of special significance, changes in the blood flow of the area could lead to impairment in sperm production [23-26].

New studies with color Doppler ultrasonography show a correlation between RI of intratesticular arteries and testicular function [27-30]. As a routinely biomarker used for male factor infertility, it has been shown that semen analysis have its own limitations. Also, most of men are uncomfortable with masturbating for semen collection, especially when performed at clinical laboratory. Therefore developing and using more convenient and more accurate diagnostic modalities deserves further investigation [31-35]. Moreover, the diagnostic value of RI by Doppler ultrasonography, as a non-invasive and available method, has not been well determined yet. Therefore, the aim of this study was to evaluate the diagnostic accuracy of RI in the capsular and intratesticular branches of the testicular artery in testicle ultrasonography of infertile men and its comparison with normal men.

## 2. Material and Methods

For this prospective case-control study, the approval of institutional ethics committee has been obtained. Cases included male patients who had been admitted to Imam Khomeini Educational Hospital in Sari, Iran, with abnormal sperm analysis (oligoasthenospermia) in their evaluation for infertility, with no identified female cause of infertility. Male patients with history of systemic diseases (e.g., diabetes mellitus and hypertension) or history of testicular disease, varicocele, testicular surgery, inguinal hernia surgery, receiving chemotherapy and radiotherapy, receiving medications that affect sperm production or motility, and receiving medication for treatment of oligoasthenospermia were excluded from this study. Control group were selected among those patients who had sperm analysis for any other reason with normal results. All patients in the control group were assessed in terms of exclusion criteria and were excluded if any of them were positive. After selecting the patients and obtaining informed consent, their demographic characteristics as well as the results of sperm analysis were recorded. All patients who meet the inclusion criteria underwent testicular sonography by two board-certified radiologist. In case of inconsistencies in the results reported by the two radiologists, re-ultrasound was performed by a radiologist with at least 5 years of experience in performing testicular ultrasonography, and the final result was recorded. E-CUBE 15 Platinum- ALPINION MEDICAL SYSTEMS ultrasound machine was used to perform ultrasonography using a 12 MHz surface probe. First, patients were placed lying in a supine position while his penis was on the abdomen. Then Doppler ultrasound was conducted to examine the blood flow of capsular and intra-testicular branches of testicular arteries in each testicle. PSV and EDV were measured for each testicle in the upper and lower poles and were used to calculate RI. Accordingly, RI was calculated using the following formula: RI = PSV/(PSV - EDV). Also, the following formula was used to calculate the testicular volume: Testicular volume = (length × width × anteroposterior diameter) × π/6 [36-38].

Statistical package for the social sciences (SPSS) version 22 software was used for data analysis. The quantitative variables were shown as Mean ± standard deviation (S.D), confidence interval (CI) and the qualitative variables were shown as frequency. Chi-square test (if needed, Fisher's exact test) was used to compare the qualitative variables. Statistical t-test was used to compare the statistical differences between the quantitative variables. Data from individual cross-tabulations were used to calculate sensitivity, specificity, positive predictive value (PPV), negative predictive value (NPV), positive likelihood ratio (PLR) and negative likelihood (NLR). P-value less than 0.05 was considered as a significance level.

## 3. Results

The total number of patients were 60 (30 healthy in control group, and 30 were in case group). The mean ages of the subjects in the control and case groups were 34.20 ± 5.77 (range: 25 to 47 years; median = 34) and 34.03 ± 5.03 (range: 26 to 44 years; median = 34) years, respectively (P = 0.906). The mean number of sperm count in the control group was 56.46 ± 15.28 million per ml (median = 60) and in the case group was 6.26 ± 4.16 million per ml (median = 5). The mean percentage of sperm motility in the control group was 42.50 ± 7.62% (median = 40%) and in the case group 12.76 ± 6.96% (median = 15%). Mean sperm count and motility were significantly higher in control group (P < 0.001).

In terms of the results of testicular ultrasonography, the mean right testicle volume in the control group was 15.40 ± 4.01 mL (median = 15 mL) and in the case group 12.15 ± 3.07 mL (median = 12.3 mL). Right testicle volume was significantly higher in control group (P = 0.001). Furthermore, the mean of left testicle volume in case group was 12.40 ± 2.84 mL (median = 12.15 mL) and in the control group was 14.25 ± 3.19 mL (median = 14.40 mL), which was significantly lower in case group (P = 0.021).

**Fig. 1:**
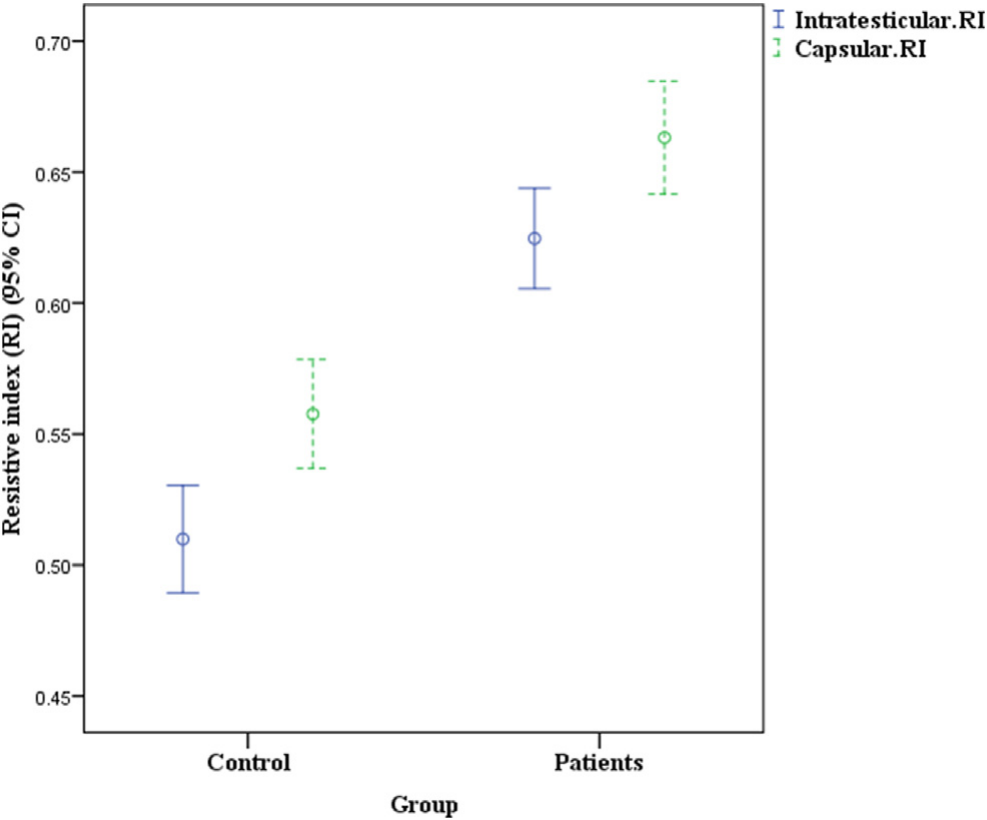
Mean RI of intratesticular and capsular arteries of the patients in case and control groups.

The results of testicular color Doppler sonography in two groups have been shown in Table and Fig. 1. As shown in Table 1, the mean RI of intratesticular and capsular arteries in the case group was significantly higher than the control group (P < 0.001).

The frequency of the normal and abnormal RI of intratesticular and capsular arteries has been shown in Table 2.

The results indicated that the frequency of abnormal RI of the intratesticular artery was significantly higher in the case group, so that the probability of having an abnormal RI with infertility was 13 times greater than of healthy subjects (OR: 13.0, 95% CI: 3.44-47.59; P < 0.001). RI measurement of intratesticular artery to diagnose male infertility showed sensitivity of 66.67% (95% CI: 47.19% −82.71%), specificity of 86.67% (95% CI: 69.28% −96.24%), PPV of 83.33% (95% CI: 65.99% −92.80%), NPV of 72.22% (95% CI: 60.60% −81.47%) and overall accuracy (OA) of 76.67% (95% CI: 63.96% −86.62%). Moreover, the PLR and NLR calculated for abnormal RI of the intratesticular artery were, 5.00 (95% CI: 1.94-12.89) and 0.38 (95% CI: 0.23-0.65); respectively. Also, the results showed that the frequency of abnormal RI of capsular artery was significantly higher in the case group, so that the likelihood of being associated with infertility was 29.57 times greater than of healthy subjects (OR: 29.57, 95% CI: 6.85-127.63; P < 0.001). Measuring RI of capsular artery to diagnose male infertility showed the sensitivity of 90% (95% CI: 73.47% −97.89%), specificity of 76.67% (95% CI: 57.72% −90.07%), PPV of 79.41% (95% CI: 66.61% −88.18%), NPV of 88.46% (95% CI: 72.02% −95.80%) and OA of 83.33% (95% CI: 71.48% −91.71%). Moreover, the PLR and NLR calculated for abnormal RI of the capsular artery were, 3.86 (95% CI: 1.99-7.46) and 0.13 (95% CI: 0.04-0.39); respectively.

## 4. Discussion

The results of this study showed that the mean sperm count, sperm motility and testicular volume were significantly higher among the healthy subjects compared to infertile men with oligoasthenospermia. The results of a study by Tijani et al. indicate that the mean testicular volume in infertile men was significantly lower that the fertile men. In addition, the reduced sperm density was associated with lower testicular volume [12]. However, Pinggera et al. did not showed any significant differences between ultrasound measured testicular volumes in healthy and infertile men [39]; possibly due to technical errors of the operator in dong sonography or sperm analysis or also quality of laboratory tools. Vinayaka et al. indicate a positive correlation between testicular size and sperm count in infertile men [40]. Another study by Manuel et al. revealed a significant positive correlation between the total sperm count and testicular volume in infertile men [41].

However, contrary to the findings of our study, Mahdavi et al. did not find a significant association between testicular size and the results of semen analysis parameters in patients with varicocele [42]. Considering that ultrasonography is highly operator-dependent technique, occurrence of technical faults may be one possible explanation for this inconsistency.

An interesting findings of this study was that infertile man had a significantly higher RI of intra-testicular and capsular arteries compared to the healthy control group. Also, RI of the intratesticular artery had a higher specificity and PPV compared to RI of capsular artery; though, RI of capsular artery had higher sensitivity and NPP compared to RI of intratesticular artery.

In a study by Biagiotti et al., showed that men with varicocele had the highest RI values compared to fertile men, but the RI values was not significantly different between various types of varicocele. Contrary to our study, RI in patients with oligoasthenospermia with indefinite cause (0.71) was significantly lower than in the control group (normal sperm analysis); with the lowest RI in patients with non-obstructive azoospermia (0.62) that was significantly lower than oligoasthenospermia with indefinite etiology. They concluded that RI and PSV are reliable markers for clinical use to diagnose men with infertility or dyspermia [43]. In another study showed that the mean RI of healthy subjects was 0.54 ± 0.05, and in patients with oligoasthenozoospermia was 0.68 ± 0.06. This study indicate that the RI of the patients with oligoasthenozoospermia was significantly higher than the healthy subjects, which was in line with the results of our study. Their results showed that RI with values >0.6 may suggestive of pathological sperm count [39]. Hillelsohn et al. examined the diagnostic value of RI > 0.06 in diagnosing sub-fertile men with spermatogenesis disorder. In their study, the patients were divided into two groups: RI of 0.6 or less (n = 49) and RI greater than 0.6 (n = 42). The results showed that RI at a threshold of greater than 0.6 had a sensitivity of 63.27% and a likelihood ratio 1.56 to predict overall sperm motility in spermatogenesis. They have concluded that intratesticular RI greater than 0.6 was associated with impaired spermatogenesis. This result supports the idea that evaluating RI using color Doppler sonography can be used as a non-invasive method to evaluate testicular function [27]. Krebs et al. assessed testicular RI in men with spinal cord injury. Mean testicular RI was calculated to be 0.69 that was significantly higher than the reported of 0.6. There was a significant positive correlation between testicular RI and sperm concentration (r = 0.81). This study showed that testicular RI in men with spinal cord injury was significantly higher than 0.6 and that its repeated measurement can be useful as an adjunct to evaluating infertility in men with spinal cord injury [44]. Except for one study [43], other studies [27, 39–44] stated RI more than 0.6 as a marker of spermatogenesis disorder among men. Like these studies, our results showed a very strong association between RI greater than 0.6 with spermatogenesis disorder in men. Moreover, the RI diagnostic cut-point value of more than 0.6 was chosen in these studies, showing that calculating RI using color Doppler sonography is a very powerful modality with acceptable sensitivity and specificity in diagnosis of oligoasthenospermia. However, it is suggested that future studies should be designed and conducted to evaluate the diagnostic value of Doppler ultrasound pulsatility index and compare its diagnostic value with Doppler ultrasound RI.

## 5. Conclusion

The results indicated that calculating RI of capsular and intratesticular branches of testicular artery using color Doppler ultrasonography has high diagnostic accuracy and may be a valuable non-invasive and simple complementary diagnostic modality in diagnosing infertile men with oligoasthenospermia.

## Figures and Tables

**Table 1:** The results of testicular color Doppler sonography in two groups.

CDU results	Control group	Case group	P-value
Right testicle volume	15.40 ± 4.01 mL	12.15 ± 3.07 mL	0.001
Left testicle volume	14.25 ± 3.19 mL	12.40 ± 2.84 mL	0.021
RI of intratesticular artery	0.509 ± 0.054	0.624 ± 0.051	<0.001
RI of capsular artery	0.557 ± 0.055	0.663 ± 0.057	<0.001

**Table 2:** Frequency of normal and abnormal RI of intratesticular and capsular arteries in two groups.

RI results	Control group	Case group	P value	
RI of intratesticular artery	Normal (0.6≥)	26 (86.7%)	10 (33.3%)	<0.001
	Abnormal (0.6 <)	4 (13.3%)	20 (66.7%)	
RI of capsular artery	Normal (0.6 ≥)	23 (76.7%)	3 (10.0%)	<0.001
	Abnormal (0.6 <)	7 (23.3%)	27 (90.0%)	
